# Pyridinium 5-[(1,3-diethyl-6-hydr­oxy-4-oxo-2-thioxo-1,2,3,4-tetra­hydro­pyrimidin-5-yl)(2-methoxy­phen­yl)meth­yl]-1,3-diethyl-4,6-dioxo-2-thioxopyrimidin-5-ide

**DOI:** 10.1107/S160053680902618X

**Published:** 2009-07-15

**Authors:** Abdullah Mohamed Asiri, Salman A. Khan, Seik Weng Ng

**Affiliations:** a Chemistry Department, Faculty of Science, King Abdul Aziz University, Jeddah, Saudi Arabia; b Department of Chemistry, University of Malaya, 50603 Kuala Lumpur, Malaysia

## Abstract

1,3-Diethyl-2-thio­barbituric acid reacts with 2-anisaldehyde to form the Michael addition product 2-anisylbis(1,3-diethyl-2-thio­barbitur-5-yl)methanate, which crystallizes as the title pyridin­ium salt, C_5_H_6_N^+^·C_24_H_29_N_4_O_5_S_2_^−^, when it reacts with the pyridine used to catalyse the reaction. There are two independent ion pairs in the crystal structure. The anion features a methine C atom connected to three six-membered rings; one of the rings carries a hydr­oxy group, which engages in hydrogen bonding with the carbonyl group belonging to another ring. The monoclinic unit cell emulates an ortho­rhom­bic unit cell, and is a twin with a minor twin component of 35%.

## Related literature

For the reaction of 1,3-diethyl-2-thio­barbituric acid with aromatic aldehydes to form the Knoevenagel and Michael products, see: Adamson *et al.* (1999 ▶).
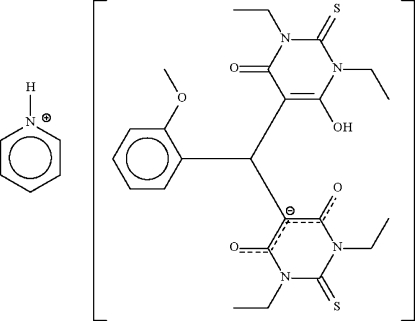


## Experimental

### 

#### Crystal data

C_5_H_6_N^+^·C_24_H_29_N_4_O_5_S_2_^−^
*M* *_r_* = 597.74
Monoclinic, 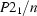

*a* = 17.3713 (3) Å
*b* = 19.8285 (3) Å
*c* = 17.3969 (2) Å
β = 90.316 (1)°
*V* = 5992.22 (15) Å^3^
*Z* = 8
Mo *K*α radiation
μ = 0.22 mm^−1^
*T* = 140 K
0.35 × 0.25 × 0.15 mm

#### Data collection

Bruker SMART APEX diffractometer
Absorption correction: multi-scan (*SADABS*; Sheldrick, 1996 ▶) *T*_min_ = 0.926, *T*_max_ = 0.967
40346 measured reflections
13375 independent reflections
11615 reflections with *I* > 2σ(*I*)
*R*_int_ = 0.047

#### Refinement

*R*[*F* ^2^ > 2σ(*F* ^2^)] = 0.068
*wR*(*F* ^2^) = 0.212
*S* = 1.02
13375 reflections
762 parameters
20 restraints
H-atom parameters constrained
Δρ_max_ = 0.96 e Å^−3^
Δρ_min_ = −0.65 e Å^−3^

### 

Data collection: *APEX2* (Bruker, 2008 ▶); cell refinement: *SAINT* (Bruker, 2008 ▶); data reduction: *SAINT*; program(s) used to solve structure: *SHELXS97* (Sheldrick, 2008 ▶); program(s) used to refine structure: *SHELXL97* (Sheldrick, 2008 ▶); molecular graphics: *X-SEED* (Barbour, 2001 ▶); software used to prepare material for publication: *publCIF* (Westrip, 2009 ▶).

## Supplementary Material

Crystal structure: contains datablocks global, I. DOI: 10.1107/S160053680902618X/xu2549sup1.cif

Structure factors: contains datablocks I. DOI: 10.1107/S160053680902618X/xu2549Isup2.hkl

Additional supplementary materials:  crystallographic information; 3D view; checkCIF report

## Figures and Tables

**Table 1:** Hydrogen-bond geometry (Å, °)

*D*—H⋯*A*	*D*—H	H⋯*A*	*D*⋯*A*	*D*—H⋯*A*
O3—H3⋯O1	0.84	1.63	2.435 (4)	159
O6—H6⋯O8	0.84	1.67	2.444 (4)	152
N11—H11⋯O2	0.88	1.93	2.721 (4)	148
N12—H12⋯O9	0.88	1.89	2.745 (6)	162

**Fig. 1.**
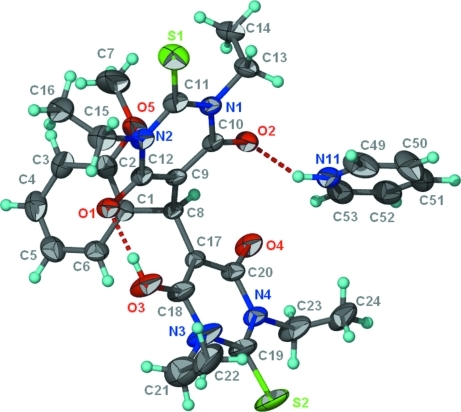
Thermal ellipsoid plot (Barbour, 2001) of one [C5H6N][C24H29N4O5S2] ion-pair at the 70% probability level; hydrogen atoms are drawn as spheres of arbitrary radius. Dash lines denote hydrogen bonds.

**Fig. 2.**
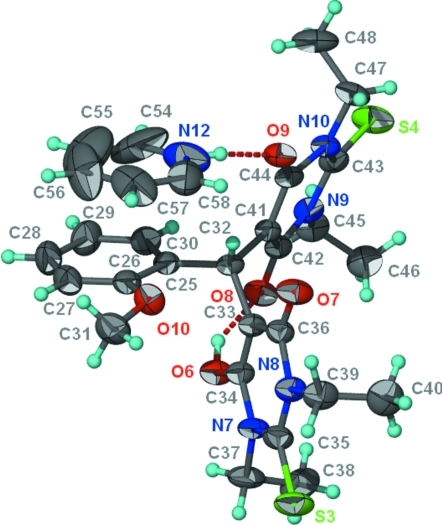
Thermal ellipsoid plot (Barbour, 2001) of second [C5H6N][C24H29N4O5S2] ion-pair at the 70% probability level; hydrogen atoms are drawn as spheres of arbitrary radius. Dash lines denote hydrogen bonds.

## supplementary crystallographic information

### Experimental

1,3-Diethyl-2-thiobarbituric acid (1.00 g, 0.005 mol) and 2-methoxy-benzaldehyde (0.68 g, 0.005 mol) were heated in ethanol (15 ml) for 3 h; several drops of pyridine were added. The progress of reaction was monitored by TLC. The solid that separated from the cooled mixture was collected and recrystallized from a methanol/chloroform mixture in yield 50%; m.p. 447 K.

### Refinement

Carbon-bound H-atoms were placed in calculated positions (C—H 0.95–0.99 Å) and were included in the refinement in the riding model approximation, with *U_iso_*(H) fixed at 1.2–1.5*U_eq_*(C).

In the anion, one of the two carbonyl groups of the diethyl-2-thiobarbitur-5-yl portion is protonated. A difference Fourier map showed the "acid" hydrogen atom approximately midway between O1 and O3 in one anion, and another approximately midway between O6 and O8 in the other. The hydroxy H-atoms were then arbitrarily placed on the O1 and O3 atoms and treated as riding (O–H 0.84 Å); their temperature factors were similarly tied.

One of the ethyl groups is disordered in the terminal carbon atom; the pair of C21–C22 and C21–C22' distances were restrained to 1.50±0.01 Å; the anistropic temperature factors of the disordered atoms were restrained to be nearly isotropic. The two pyridinium rings were restrained to within 0.01 Å of planarity.

The monoclinic unit cell emulates an orthorhombic unit cell as the β angle is nearly a right angle. The structure was refined by using the twin law (-1 0 0 0 - 1 0 0 0 1). The twin component refined to 35%.

### Crystal data

**Table:**

C_5_H_6_N^+^·C_24_H_29_N_4_O_5_S_2_^−^	*F*(000) = 2528
*M**_r_* = 597.74	*D*_x_ = 1.325 Mg m^−^^3^
Monoclinic, *P*2_1_/*n*	Mo *K*α radiation, λ = 0.71073 Å
Hall symbol: -P 2yn	Cell parameters from 9952 reflections
*a* = 17.3713 (3) Å	θ = 2.3–28.3°
*b* = 19.8285 (3) Å	µ = 0.22 mm^−^^1^
*c* = 17.3969 (2) Å	*T* = 140 K
β = 90.316 (1)°	Block, yellow
*V* = 5992.22 (15) Å^3^	0.35 × 0.25 × 0.15 mm
*Z* = 8	

### Data collection

**Table:**

Bruker SMART APEX diffractometer	13375 independent reflections
Radiation source: fine-focus sealed tube	11615 reflections with *I* > 2σ(*I*)
graphite	*R*_int_ = 0.047
ω scans	θ_max_ = 27.5°, θ_min_ = 1.0°
Absorption correction: multi-scan (*SADABS* (Sheldrick, 1996)	*h* = −22→20
*T*_min_ = 0.926, *T*_max_ = 0.967	*k* = −25→25
40346 measured reflections	*l* = −22→22

### Refinement

**Table:**

Refinement on *F*^2^	Primary atom site location: structure-invariant direct methods
Least-squares matrix: full	Secondary atom site location: difference Fourier map
*R*[*F*^2^ > 2σ(*F*^2^)] = 0.068	Hydrogen site location: inferred from neighbouring sites
*wR*(*F*^2^) = 0.212	H-atom parameters constrained
*S* = 1.02	*w* = 1/[σ^2^(*F*_o_^2^) + (0.1371*P*)^2^ + 5.8594*P*] where *P* = (*F*_o_^2^ + 2*F*_c_^2^)/3
13375 reflections	(Δ/σ)_max_ = 0.001
762 parameters	Δρ_max_ = 0.96 e Å^−^^3^
20 restraints	Δρ_min_ = −0.65 e Å^−^^3^

### Fractional atomic coordinates and isotropic or equivalent isotropic displacement parameters (Å^2^)

**Table:**

	*x*	*y*	*z*	*U*_iso_*/*U*_eq_	Occ. (<1)
S1	0.89771 (8)	0.05444 (5)	0.52087 (6)	0.0463 (3)	
S2	0.82015 (11)	0.54186 (6)	0.28660 (8)	0.0731 (5)	
S3	0.58599 (8)	1.00100 (5)	0.73444 (7)	0.0494 (3)	
S4	0.66510 (8)	0.48083 (5)	0.59111 (7)	0.0499 (3)	
O1	1.02160 (16)	0.27848 (12)	0.49436 (13)	0.0314 (5)	
O2	0.90126 (16)	0.18218 (12)	0.27730 (13)	0.0311 (5)	
O3	0.9696 (2)	0.38554 (15)	0.44929 (16)	0.0530 (9)	
H3	0.9894	0.3471	0.4535	0.080*	
O4	0.94268 (19)	0.33612 (14)	0.18598 (14)	0.0397 (7)	
O5	1.09713 (18)	0.17784 (14)	0.3064 (2)	0.0463 (7)	
O6	0.47753 (17)	0.77199 (13)	0.75929 (14)	0.0337 (6)	
H6	0.4818	0.7323	0.7430	0.051*	
O7	0.56297 (19)	0.84240 (13)	0.51601 (15)	0.0394 (6)	
O8	0.53137 (17)	0.66121 (13)	0.72942 (14)	0.0346 (6)	
O9	0.53358 (17)	0.66864 (13)	0.45855 (14)	0.0344 (6)	
O10	0.37221 (17)	0.83328 (13)	0.56120 (17)	0.0367 (6)	
N1	0.90240 (18)	0.12805 (14)	0.39263 (16)	0.0267 (6)	
N2	0.96084 (19)	0.17596 (14)	0.50060 (15)	0.0285 (6)	
N3	0.9043 (3)	0.4538 (2)	0.3675 (2)	0.0652 (15)	
N4	0.8902 (2)	0.43076 (15)	0.23793 (17)	0.0341 (7)	
N7	0.5287 (2)	0.87620 (15)	0.74018 (17)	0.0321 (7)	
N8	0.5688 (2)	0.91224 (15)	0.61993 (18)	0.0324 (7)	
N9	0.58764 (19)	0.57995 (14)	0.65831 (17)	0.0300 (6)	
N10	0.59868 (19)	0.58880 (14)	0.52586 (16)	0.0289 (6)	
N11	0.8163 (2)	0.23051 (15)	0.1576 (2)	0.0446 (9)	
H11	0.8513	0.2305	0.1943	0.053*	
N12	0.4054 (4)	0.7341 (3)	0.4035 (3)	0.084 (2)	
H12	0.4506	0.7154	0.4118	0.101*	
C1	1.0903 (2)	0.29287 (17)	0.33653 (18)	0.0273 (7)	
C2	1.1362 (2)	0.23530 (19)	0.3249 (2)	0.0335 (8)	
C3	1.2161 (2)	0.2388 (2)	0.3299 (2)	0.0385 (8)	
H3A	1.2463	0.1995	0.3219	0.046*	
C4	1.2516 (2)	0.2999 (2)	0.3467 (2)	0.0429 (9)	
H4	1.3061	0.3025	0.3504	0.051*	
C5	1.2076 (3)	0.3568 (2)	0.3579 (2)	0.0423 (9)	
H5	1.2317	0.3986	0.3695	0.051*	
C6	1.1281 (2)	0.35302 (18)	0.3523 (2)	0.0339 (8)	
H6A	1.0985	0.3928	0.3595	0.041*	
C7	1.1359 (4)	0.1150 (3)	0.3186 (6)	0.093 (3)	
H7A	1.0978	0.0791	0.3254	0.140*	
H7B	1.1683	0.1182	0.3648	0.140*	
H7C	1.1681	0.1047	0.2741	0.140*	
C8	1.0031 (2)	0.28629 (15)	0.32585 (17)	0.0230 (6)	
H8	0.9974	0.2653	0.2738	0.028*	
C9	0.9680 (2)	0.23466 (15)	0.37963 (18)	0.0238 (6)	
C10	0.9232 (2)	0.18309 (15)	0.34538 (18)	0.0247 (6)	
C11	0.9218 (2)	0.12219 (17)	0.46899 (19)	0.0297 (7)	
C12	0.9848 (2)	0.23158 (17)	0.45709 (18)	0.0263 (6)	
C13	0.8634 (2)	0.07179 (18)	0.3524 (2)	0.0333 (7)	
H13A	0.8271	0.0901	0.3135	0.040*	
H13B	0.8335	0.0450	0.3898	0.040*	
C14	0.9222 (3)	0.02676 (19)	0.3132 (2)	0.0389 (9)	
H14A	0.8955	−0.0102	0.2867	0.058*	
H14B	0.9575	0.0082	0.3519	0.058*	
H14C	0.9513	0.0533	0.2758	0.058*	
C15	0.9865 (3)	0.1733 (2)	0.5822 (2)	0.0409 (9)	
H15A	0.9481	0.1482	0.6128	0.049*	
H15B	0.9898	0.2197	0.6030	0.049*	
C16	1.0634 (3)	0.1396 (2)	0.5901 (3)	0.0474 (10)	
H16A	1.0792	0.1396	0.6442	0.071*	
H16B	1.1015	0.1642	0.5596	0.071*	
H16C	1.0597	0.0931	0.5716	0.071*	
C17	0.9605 (2)	0.35256 (16)	0.31862 (18)	0.0250 (6)	
C18	0.9464 (3)	0.39456 (19)	0.3793 (2)	0.0406 (10)	
C19	0.8739 (3)	0.4722 (2)	0.2978 (3)	0.0475 (11)	
C20	0.9334 (2)	0.37039 (17)	0.24468 (19)	0.0283 (7)	
C21	0.9036 (4)	0.5027 (3)	0.4319 (3)	0.0679 (16)	
H21A	0.9492	0.4981	0.4658	0.081*	0.85
H21B	0.8990	0.5499	0.4138	0.081*	0.85
H21C	0.9521	0.4880	0.4568	0.081*	0.15
H21D	0.9191	0.5429	0.4019	0.081*	0.15
C22	0.8332 (4)	0.4788 (4)	0.4685 (6)	0.082 (2)	0.85
H22A	0.8187	0.5100	0.5097	0.122*	0.85
H22B	0.8421	0.4338	0.4902	0.122*	0.85
H22C	0.7917	0.4765	0.4303	0.122*	0.85
C22'	0.8718 (16)	0.5387 (14)	0.4979 (12)	0.056 (7)	0.15
H22D	0.8471	0.5065	0.5326	0.084*	0.15
H22E	0.8337	0.5717	0.4800	0.084*	0.15
H22F	0.9133	0.5622	0.5254	0.084*	0.15
C23	0.8631 (3)	0.4488 (2)	0.1597 (2)	0.0528 (12)	
H23A	0.8977	0.4281	0.1213	0.063*	
H23B	0.8656	0.4983	0.1533	0.063*	
C24	0.7825 (4)	0.4255 (3)	0.1447 (3)	0.0718 (19)	
H24A	0.7685	0.4349	0.0911	0.108*	
H24B	0.7472	0.4495	0.1789	0.108*	
H24C	0.7789	0.3769	0.1544	0.108*	
C25	0.3964 (2)	0.72617 (16)	0.61215 (19)	0.0274 (6)	
C26	0.3433 (2)	0.77706 (17)	0.5956 (2)	0.0307 (7)	
C27	0.2648 (3)	0.7681 (2)	0.6107 (3)	0.0415 (9)	
H27	0.2292	0.8034	0.6006	0.050*	
C28	0.2396 (3)	0.7076 (2)	0.6406 (3)	0.0476 (10)	
H28	0.1864	0.7014	0.6508	0.057*	
C29	0.2903 (3)	0.6565 (2)	0.6557 (3)	0.0465 (10)	
H29	0.2723	0.6148	0.6755	0.056*	
C30	0.3692 (2)	0.66590 (19)	0.6417 (2)	0.0368 (8)	
H30	0.4044	0.6304	0.6527	0.044*	
C31	0.3244 (3)	0.8913 (2)	0.5552 (3)	0.0462 (10)	
H31A	0.3549	0.9294	0.5364	0.069*	
H31B	0.3035	0.9022	0.6059	0.069*	
H31C	0.2821	0.8822	0.5193	0.069*	
C32	0.4807 (2)	0.73835 (15)	0.59246 (18)	0.0247 (6)	
H32	0.4795	0.7512	0.5369	0.030*	
C33	0.5154 (2)	0.80019 (16)	0.63262 (19)	0.0264 (6)	
C34	0.5065 (2)	0.81388 (17)	0.7090 (2)	0.0282 (7)	
C35	0.5592 (2)	0.92679 (18)	0.6963 (2)	0.0336 (8)	
C36	0.5499 (2)	0.84948 (16)	0.5848 (2)	0.0291 (7)	
C37	0.5267 (3)	0.8834 (2)	0.8252 (2)	0.0442 (10)	
H37A	0.4827	0.8576	0.8459	0.053*	
H37B	0.5194	0.9315	0.8388	0.053*	
C38	0.6009 (3)	0.8578 (3)	0.8608 (2)	0.0582 (13)	
H38A	0.5989	0.8635	0.9168	0.087*	
H38B	0.6444	0.8835	0.8404	0.087*	
H38C	0.6074	0.8100	0.8485	0.087*	
C39	0.6062 (3)	0.96227 (18)	0.5699 (2)	0.0397 (9)	
H39A	0.5914	1.0083	0.5862	0.048*	
H39B	0.5886	0.9558	0.5162	0.048*	
C40	0.6927 (3)	0.9547 (2)	0.5743 (3)	0.0515 (11)	
H40A	0.7170	0.9893	0.5423	0.077*	
H40B	0.7073	0.9099	0.5556	0.077*	
H40C	0.7098	0.9600	0.6277	0.077*	
C41	0.52967 (19)	0.67496 (16)	0.59453 (19)	0.0252 (6)	
C42	0.5485 (2)	0.64163 (16)	0.6621 (2)	0.0273 (7)	
C43	0.6146 (2)	0.55326 (16)	0.5918 (2)	0.0316 (7)	
C44	0.5510 (2)	0.64639 (16)	0.52333 (19)	0.0278 (7)	
C45	0.5976 (3)	0.54224 (18)	0.7313 (2)	0.0370 (8)	
H45A	0.5955	0.4933	0.7203	0.044*	
H45B	0.5541	0.5531	0.7658	0.044*	
C46	0.6722 (3)	0.5579 (2)	0.7726 (2)	0.0418 (9)	
H46A	0.6724	0.5360	0.8231	0.063*	
H46B	0.6772	0.6068	0.7791	0.063*	
H46C	0.7155	0.5411	0.7422	0.063*	
C47	0.6276 (3)	0.56422 (19)	0.4514 (2)	0.0386 (9)	
H47A	0.6781	0.5420	0.4595	0.046*	
H47B	0.6356	0.6031	0.4167	0.046*	
C48	0.5727 (4)	0.5146 (2)	0.4132 (3)	0.0545 (13)	
H48A	0.5943	0.4997	0.3642	0.082*	
H48B	0.5230	0.5366	0.4040	0.082*	
H48C	0.5654	0.4755	0.4469	0.082*	
C49	0.7469 (4)	0.20668 (18)	0.1732 (3)	0.0599 (15)	
H49	0.7353	0.1905	0.2232	0.072*	
C50	0.6911 (3)	0.2057 (2)	0.1152 (3)	0.0561 (12)	
H50	0.6409	0.1890	0.1254	0.067*	
C51	0.7094 (3)	0.22889 (19)	0.0435 (3)	0.0438 (10)	
H51	0.6723	0.2281	0.0032	0.053*	
C52	0.7817 (3)	0.2532 (2)	0.0305 (2)	0.0426 (9)	
H52	0.7950	0.2693	−0.0191	0.051*	
C53	0.8354 (3)	0.25457 (19)	0.0886 (2)	0.0406 (9)	
H53	0.8854	0.2723	0.0799	0.049*	
C54	0.3430 (5)	0.7006 (3)	0.4243 (4)	0.100 (3)	
H54	0.3471	0.6573	0.4472	0.120*	
C55	0.2742 (5)	0.7290 (4)	0.4123 (4)	0.099 (3)	
H55	0.2288	0.7057	0.4271	0.119*	
C56	0.2688 (4)	0.7902 (4)	0.3796 (3)	0.089 (2)	
H56	0.2193	0.8092	0.3710	0.107*	
C57	0.3338 (4)	0.8264 (3)	0.3579 (3)	0.0691 (17)	
H57	0.3298	0.8701	0.3359	0.083*	
C58	0.4028 (3)	0.7964 (3)	0.3698 (3)	0.0607 (13)	
H58	0.4490	0.8184	0.3549	0.073*	

### Atomic displacement parameters (Å^2^)

**Table:**

	*U*^11^	*U*^22^	*U*^33^	*U*^12^	*U*^13^	*U*^23^
S1	0.0661 (7)	0.0383 (5)	0.0346 (5)	−0.0118 (5)	0.0028 (5)	0.0155 (4)
S2	0.1245 (13)	0.0392 (6)	0.0553 (7)	0.0464 (7)	−0.0402 (8)	−0.0121 (5)
S3	0.0697 (8)	0.0273 (4)	0.0513 (6)	−0.0118 (5)	0.0103 (5)	−0.0147 (4)
S4	0.0762 (8)	0.0233 (4)	0.0501 (6)	0.0137 (5)	−0.0059 (6)	−0.0046 (4)
O1	0.0467 (15)	0.0252 (11)	0.0223 (11)	0.0039 (10)	−0.0068 (10)	0.0003 (9)
O2	0.0456 (15)	0.0245 (11)	0.0232 (11)	−0.0016 (10)	−0.0073 (10)	0.0018 (8)
O3	0.096 (3)	0.0389 (15)	0.0241 (12)	0.0336 (17)	−0.0160 (14)	−0.0062 (11)
O4	0.0601 (19)	0.0378 (14)	0.0212 (11)	0.0146 (13)	−0.0043 (11)	0.0023 (10)
O5	0.0366 (15)	0.0339 (14)	0.068 (2)	0.0064 (12)	−0.0075 (14)	−0.0204 (13)
O6	0.0484 (16)	0.0261 (12)	0.0268 (11)	−0.0051 (11)	0.0065 (11)	−0.0014 (9)
O7	0.0605 (18)	0.0311 (13)	0.0268 (12)	−0.0067 (12)	0.0094 (12)	−0.0013 (10)
O8	0.0502 (16)	0.0310 (12)	0.0225 (11)	0.0045 (11)	0.0012 (11)	0.0015 (9)
O9	0.0462 (16)	0.0299 (12)	0.0271 (12)	0.0037 (11)	−0.0029 (11)	−0.0037 (9)
O10	0.0408 (15)	0.0279 (12)	0.0415 (15)	0.0048 (11)	0.0033 (11)	0.0064 (10)
N1	0.0329 (15)	0.0221 (12)	0.0251 (13)	0.0022 (11)	−0.0009 (11)	0.0032 (10)
N2	0.0409 (17)	0.0268 (13)	0.0177 (12)	0.0037 (12)	0.0009 (11)	0.0038 (10)
N3	0.119 (4)	0.045 (2)	0.0319 (18)	0.047 (2)	−0.023 (2)	−0.0119 (15)
N4	0.0509 (19)	0.0251 (13)	0.0263 (14)	0.0072 (13)	−0.0088 (13)	0.0046 (11)
N7	0.0443 (18)	0.0262 (14)	0.0260 (14)	−0.0064 (13)	0.0052 (13)	−0.0079 (11)
N8	0.0421 (18)	0.0213 (13)	0.0339 (15)	−0.0045 (12)	0.0026 (13)	−0.0001 (11)
N9	0.0384 (16)	0.0208 (12)	0.0307 (14)	−0.0031 (12)	−0.0032 (12)	0.0038 (11)
N10	0.0379 (16)	0.0214 (12)	0.0275 (13)	−0.0010 (12)	0.0013 (12)	−0.0029 (10)
N11	0.061 (2)	0.0288 (16)	0.0438 (19)	0.0070 (16)	−0.0219 (17)	−0.0060 (13)
N12	0.091 (4)	0.079 (4)	0.082 (4)	0.052 (3)	−0.039 (3)	−0.038 (3)
C1	0.0353 (18)	0.0274 (15)	0.0192 (13)	−0.0009 (14)	−0.0025 (13)	0.0035 (11)
C2	0.039 (2)	0.0337 (18)	0.0274 (16)	0.0002 (16)	−0.0041 (14)	−0.0029 (13)
C3	0.0320 (19)	0.045 (2)	0.0382 (19)	0.0049 (17)	−0.0013 (15)	0.0013 (16)
C4	0.034 (2)	0.055 (2)	0.039 (2)	−0.0089 (18)	−0.0046 (17)	0.0127 (18)
C5	0.047 (2)	0.038 (2)	0.041 (2)	−0.0144 (18)	−0.0060 (18)	0.0092 (16)
C6	0.044 (2)	0.0246 (16)	0.0327 (17)	−0.0030 (15)	−0.0035 (15)	0.0066 (13)
C7	0.065 (4)	0.041 (3)	0.172 (8)	0.017 (3)	−0.026 (4)	−0.039 (4)
C8	0.0313 (17)	0.0192 (14)	0.0184 (13)	0.0021 (12)	−0.0027 (12)	0.0019 (10)
C9	0.0311 (17)	0.0180 (13)	0.0225 (14)	0.0028 (12)	−0.0005 (12)	0.0036 (11)
C10	0.0297 (16)	0.0211 (14)	0.0232 (14)	0.0044 (12)	0.0008 (12)	0.0031 (11)
C11	0.0379 (19)	0.0270 (15)	0.0241 (15)	0.0031 (14)	0.0049 (13)	0.0048 (12)
C12	0.0325 (17)	0.0248 (15)	0.0218 (14)	0.0049 (13)	0.0008 (12)	0.0011 (11)
C13	0.041 (2)	0.0240 (15)	0.0353 (18)	−0.0060 (14)	−0.0004 (15)	0.0011 (13)
C14	0.052 (2)	0.0257 (17)	0.039 (2)	−0.0021 (16)	0.0042 (17)	−0.0019 (14)
C15	0.068 (3)	0.0378 (19)	0.0172 (14)	0.0006 (19)	−0.0033 (16)	0.0067 (13)
C16	0.067 (3)	0.037 (2)	0.037 (2)	0.000 (2)	−0.016 (2)	0.0117 (17)
C17	0.0329 (17)	0.0201 (14)	0.0220 (14)	0.0020 (13)	−0.0044 (12)	0.0029 (11)
C18	0.065 (3)	0.0295 (18)	0.0269 (17)	0.0176 (18)	−0.0107 (17)	−0.0022 (13)
C19	0.076 (3)	0.0266 (17)	0.040 (2)	0.0196 (19)	−0.019 (2)	−0.0036 (15)
C20	0.0382 (19)	0.0224 (15)	0.0242 (15)	0.0027 (13)	0.0009 (13)	0.0042 (12)
C21	0.092 (4)	0.061 (3)	0.051 (3)	0.021 (3)	−0.009 (3)	−0.007 (2)
C22	0.061 (4)	0.062 (4)	0.122 (6)	0.004 (3)	−0.022 (4)	0.036 (4)
C22'	0.050 (10)	0.047 (10)	0.070 (11)	−0.015 (8)	−0.033 (8)	0.018 (8)
C23	0.085 (4)	0.044 (2)	0.0287 (19)	0.024 (2)	−0.009 (2)	0.0094 (16)
C24	0.103 (5)	0.058 (3)	0.054 (3)	0.044 (3)	−0.047 (3)	−0.022 (2)
C25	0.0302 (17)	0.0253 (15)	0.0266 (15)	−0.0026 (13)	0.0019 (13)	−0.0035 (11)
C26	0.0344 (18)	0.0272 (16)	0.0305 (16)	−0.0025 (14)	0.0019 (14)	−0.0040 (13)
C27	0.037 (2)	0.0348 (19)	0.053 (2)	0.0056 (16)	0.0042 (18)	−0.0061 (17)
C28	0.032 (2)	0.043 (2)	0.068 (3)	−0.0055 (17)	0.011 (2)	−0.006 (2)
C29	0.042 (2)	0.036 (2)	0.062 (3)	−0.0127 (18)	0.006 (2)	0.0050 (19)
C30	0.039 (2)	0.0252 (16)	0.046 (2)	−0.0035 (15)	0.0031 (17)	−0.0004 (15)
C31	0.053 (3)	0.0295 (19)	0.056 (2)	0.0086 (18)	0.003 (2)	−0.0013 (17)
C32	0.0327 (17)	0.0197 (13)	0.0218 (13)	−0.0017 (12)	−0.0009 (12)	−0.0008 (11)
C33	0.0327 (17)	0.0211 (14)	0.0255 (15)	−0.0011 (13)	0.0006 (13)	−0.0004 (11)
C34	0.0341 (18)	0.0240 (15)	0.0265 (15)	−0.0019 (13)	0.0010 (13)	−0.0021 (12)
C35	0.040 (2)	0.0231 (15)	0.0375 (19)	−0.0006 (14)	0.0035 (15)	−0.0054 (13)
C36	0.0343 (17)	0.0222 (14)	0.0309 (16)	−0.0004 (13)	0.0031 (14)	−0.0022 (13)
C37	0.062 (3)	0.042 (2)	0.0286 (18)	−0.013 (2)	0.0146 (18)	−0.0141 (15)
C38	0.064 (3)	0.084 (4)	0.0262 (18)	−0.021 (3)	−0.005 (2)	−0.006 (2)
C39	0.054 (2)	0.0221 (16)	0.043 (2)	−0.0058 (16)	0.0038 (18)	0.0031 (14)
C40	0.060 (3)	0.039 (2)	0.055 (3)	−0.008 (2)	0.007 (2)	0.0080 (19)
C41	0.0299 (17)	0.0216 (14)	0.0243 (14)	−0.0011 (12)	0.0011 (12)	−0.0023 (11)
C42	0.0322 (17)	0.0213 (14)	0.0285 (15)	−0.0031 (13)	0.0004 (13)	−0.0010 (12)
C43	0.0380 (19)	0.0187 (14)	0.0380 (18)	−0.0005 (13)	−0.0047 (15)	−0.0018 (13)
C44	0.0356 (18)	0.0215 (14)	0.0264 (15)	−0.0047 (13)	−0.0007 (13)	−0.0033 (12)
C45	0.050 (2)	0.0265 (16)	0.0343 (18)	−0.0021 (16)	−0.0004 (17)	0.0104 (14)
C46	0.052 (2)	0.041 (2)	0.0321 (18)	0.0006 (18)	−0.0065 (17)	0.0075 (15)
C47	0.053 (2)	0.0277 (17)	0.0350 (18)	0.0056 (16)	0.0085 (17)	−0.0076 (14)
C48	0.093 (4)	0.035 (2)	0.035 (2)	−0.005 (2)	−0.001 (2)	−0.0122 (17)
C49	0.106 (5)	0.036 (2)	0.038 (2)	−0.018 (3)	−0.007 (3)	0.0108 (18)
C50	0.055 (3)	0.054 (3)	0.059 (3)	−0.027 (2)	0.002 (2)	0.004 (2)
C51	0.053 (3)	0.0336 (19)	0.044 (2)	−0.0022 (18)	−0.0179 (19)	0.0011 (16)
C52	0.060 (3)	0.0358 (19)	0.0323 (18)	0.0000 (19)	0.0007 (18)	−0.0041 (15)
C53	0.044 (2)	0.0368 (19)	0.041 (2)	0.0002 (17)	0.0016 (18)	−0.0145 (16)
C54	0.127 (7)	0.051 (3)	0.122 (7)	−0.003 (4)	−0.070 (6)	−0.022 (4)
C55	0.098 (6)	0.118 (7)	0.081 (5)	−0.023 (5)	−0.042 (4)	0.022 (5)
C56	0.056 (4)	0.142 (7)	0.069 (4)	0.038 (4)	−0.008 (3)	0.032 (4)
C57	0.092 (4)	0.083 (4)	0.032 (2)	0.030 (4)	−0.002 (3)	0.011 (2)
C58	0.052 (3)	0.086 (4)	0.044 (2)	−0.001 (3)	0.000 (2)	−0.012 (2)

### Geometric parameters (Å, °)

**Table:**

S1—C11	1.673 (3)	C21—C22	1.461 (8)
S2—C19	1.679 (4)	C21—C22'	1.464 (10)
S3—C35	1.678 (4)	C21—H21A	0.9900
S4—C43	1.683 (4)	C21—H21B	0.9900
O1—C12	1.300 (4)	C21—H21C	0.9900
O2—C10	1.242 (4)	C21—H21D	0.9900
O3—C18	1.294 (4)	C22—H22A	0.9800
O3—H3	0.8400	C22—H22B	0.9800
O4—C20	1.238 (4)	C22—H22C	0.9800
O5—C2	1.364 (5)	C22'—H22D	0.9800
O5—C7	1.431 (6)	C22'—H22E	0.9800
O6—C34	1.309 (4)	C22'—H22F	0.9800
O6—H6	0.8400	C23—C24	1.495 (9)
O7—C36	1.227 (4)	C23—H23A	0.9900
O8—C42	1.271 (4)	C23—H23B	0.9900
O9—C44	1.246 (4)	C24—H24A	0.9800
O10—C26	1.362 (4)	C24—H24B	0.9800
O10—C31	1.422 (5)	C24—H24C	0.9800
N1—C11	1.374 (4)	C25—C30	1.385 (5)
N1—C10	1.415 (4)	C25—C26	1.397 (5)
N1—C13	1.480 (4)	C25—C32	1.524 (5)
N2—C11	1.376 (5)	C26—C27	1.402 (6)
N2—C12	1.402 (4)	C27—C28	1.378 (6)
N2—C15	1.487 (4)	C27—H27	0.9500
N3—C19	1.370 (5)	C28—C29	1.368 (7)
N3—C18	1.397 (5)	C28—H28	0.9500
N3—C21	1.482 (6)	C29—C30	1.406 (6)
N4—C19	1.358 (5)	C29—H29	0.9500
N4—C20	1.417 (4)	C30—H30	0.9500
N4—C23	1.482 (5)	C31—H31A	0.9800
N7—C35	1.369 (5)	C31—H31B	0.9800
N7—C34	1.402 (4)	C31—H31C	0.9800
N7—C37	1.486 (5)	C32—C41	1.518 (4)
N8—C35	1.371 (5)	C32—C33	1.533 (4)
N8—C36	1.424 (4)	C32—H32	1.0000
N8—C39	1.473 (5)	C33—C34	1.367 (5)
N9—C43	1.358 (5)	C33—C36	1.419 (5)
N9—C42	1.401 (4)	C37—C38	1.515 (8)
N9—C45	1.484 (4)	C37—H37A	0.9900
N10—C43	1.373 (5)	C37—H37B	0.9900
N10—C44	1.411 (5)	C38—H38A	0.9800
N10—C47	1.475 (5)	C38—H38B	0.9800
N11—C49	1.324 (7)	C38—H38C	0.9800
N11—C53	1.335 (6)	C39—C40	1.512 (7)
N11—H11	0.8800	C39—H39A	0.9900
N12—C54	1.322 (11)	C39—H39B	0.9900
N12—C58	1.369 (9)	C40—H40A	0.9800
N12—H12	0.8800	C40—H40B	0.9800
C1—C6	1.388 (5)	C40—H40C	0.9800
C1—C2	1.408 (5)	C41—C42	1.385 (5)
C1—C8	1.530 (5)	C41—C44	1.413 (5)
C2—C3	1.391 (6)	C45—C46	1.511 (6)
C3—C4	1.390 (6)	C45—H45A	0.9900
C3—H3A	0.9500	C45—H45B	0.9900
C4—C5	1.377 (7)	C46—H46A	0.9800
C4—H4	0.9500	C46—H46B	0.9800
C5—C6	1.386 (6)	C46—H46C	0.9800
C5—H5	0.9500	C47—C48	1.521 (6)
C6—H6A	0.9500	C47—H47A	0.9900
C7—H7A	0.9800	C47—H47B	0.9900
C7—H7B	0.9800	C48—H48A	0.9800
C7—H7C	0.9800	C48—H48B	0.9800
C8—C17	1.513 (4)	C48—H48C	0.9800
C8—C9	1.517 (4)	C49—C50	1.395 (8)
C8—H8	1.0000	C49—H49	0.9500
C9—C12	1.379 (4)	C50—C51	1.370 (7)
C9—C10	1.414 (5)	C50—H50	0.9500
C13—C14	1.521 (6)	C51—C52	1.366 (7)
C13—H13A	0.9900	C51—H51	0.9500
C13—H13B	0.9900	C52—C53	1.372 (6)
C14—H14A	0.9800	C52—H52	0.9500
C14—H14B	0.9800	C53—H53	0.9500
C14—H14C	0.9800	C54—C55	1.337 (12)
C15—C16	1.498 (7)	C54—H54	0.9500
C15—H15A	0.9900	C55—C56	1.343 (11)
C15—H15B	0.9900	C55—H55	0.9500
C16—H16A	0.9800	C56—C57	1.392 (11)
C16—H16B	0.9800	C56—H56	0.9500
C16—H16C	0.9800	C57—C58	1.354 (9)
C17—C18	1.367 (5)	C57—H57	0.9500
C17—C20	1.412 (4)	C58—H58	0.9500
			
C18—O3—H3	109.5	C24—C23—H23B	109.2
C2—O5—C7	117.3 (4)	H23A—C23—H23B	107.9
C34—O6—H6	109.5	C23—C24—H24A	109.5
C26—O10—C31	118.5 (3)	C23—C24—H24B	109.5
C11—N1—C10	124.4 (3)	H24A—C24—H24B	109.5
C11—N1—C13	120.2 (3)	C23—C24—H24C	109.5
C10—N1—C13	115.1 (3)	H24A—C24—H24C	109.5
C11—N2—C12	122.7 (3)	H24B—C24—H24C	109.5
C11—N2—C15	119.9 (3)	C30—C25—C26	118.3 (3)
C12—N2—C15	117.1 (3)	C30—C25—C32	123.4 (3)
C19—N3—C18	123.5 (3)	C26—C25—C32	118.3 (3)
C19—N3—C21	119.3 (4)	O10—C26—C25	115.9 (3)
C18—N3—C21	116.5 (4)	O10—C26—C27	123.2 (4)
C19—N4—C20	124.0 (3)	C25—C26—C27	120.8 (3)
C19—N4—C23	119.5 (3)	C28—C27—C26	119.5 (4)
C20—N4—C23	116.4 (3)	C28—C27—H27	120.3
C35—N7—C34	122.5 (3)	C26—C27—H27	120.3
C35—N7—C37	119.7 (3)	C29—C28—C27	120.8 (4)
C34—N7—C37	117.5 (3)	C29—C28—H28	119.6
C35—N8—C36	124.8 (3)	C27—C28—H28	119.6
C35—N8—C39	119.2 (3)	C28—C29—C30	119.7 (4)
C36—N8—C39	115.9 (3)	C28—C29—H29	120.2
C43—N9—C42	123.3 (3)	C30—C29—H29	120.2
C43—N9—C45	119.6 (3)	C25—C30—C29	120.9 (4)
C42—N9—C45	117.0 (3)	C25—C30—H30	119.5
C43—N10—C44	123.8 (3)	C29—C30—H30	119.5
C43—N10—C47	119.7 (3)	O10—C31—H31A	109.5
C44—N10—C47	116.3 (3)	O10—C31—H31B	109.5
C49—N11—C53	122.9 (4)	H31A—C31—H31B	109.5
C49—N11—H11	118.5	O10—C31—H31C	109.5
C53—N11—H11	118.5	H31A—C31—H31C	109.5
C54—N12—C58	123.1 (6)	H31B—C31—H31C	109.5
C54—N12—H12	118.5	C41—C32—C25	113.7 (3)
C58—N12—H12	118.5	C41—C32—C33	115.6 (3)
C6—C1—C2	117.2 (3)	C25—C32—C33	113.6 (3)
C6—C1—C8	124.3 (3)	C41—C32—H32	104.1
C2—C1—C8	118.3 (3)	C25—C32—H32	104.1
O5—C2—C3	123.4 (4)	C33—C32—H32	104.1
O5—C2—C1	115.5 (3)	C34—C33—C36	119.0 (3)
C3—C2—C1	121.1 (4)	C34—C33—C32	123.8 (3)
C4—C3—C2	119.9 (4)	C36—C33—C32	116.7 (3)
C4—C3—H3A	120.1	O6—C34—C33	124.7 (3)
C2—C3—H3A	120.1	O6—C34—N7	114.0 (3)
C5—C4—C3	119.8 (4)	C33—C34—N7	121.2 (3)
C5—C4—H4	120.1	N7—C35—N8	115.8 (3)
C3—C4—H4	120.1	N7—C35—S3	122.0 (3)
C4—C5—C6	120.0 (4)	N8—C35—S3	122.2 (3)
C4—C5—H5	120.0	O7—C36—C33	125.0 (3)
C6—C5—H5	120.0	O7—C36—N8	118.4 (3)
C5—C6—C1	122.0 (4)	C33—C36—N8	116.6 (3)
C5—C6—H6A	119.0	N7—C37—C38	110.5 (4)
C1—C6—H6A	119.0	N7—C37—H37A	109.5
O5—C7—H7A	109.5	C38—C37—H37A	109.5
O5—C7—H7B	109.5	N7—C37—H37B	109.5
H7A—C7—H7B	109.5	C38—C37—H37B	109.5
O5—C7—H7C	109.5	H37A—C37—H37B	108.1
H7A—C7—H7C	109.5	C37—C38—H38A	109.5
H7B—C7—H7C	109.5	C37—C38—H38B	109.5
C17—C8—C9	116.0 (3)	H38A—C38—H38B	109.5
C17—C8—C1	114.8 (3)	C37—C38—H38C	109.5
C9—C8—C1	112.6 (3)	H38A—C38—H38C	109.5
C17—C8—H8	103.8	H38B—C38—H38C	109.5
C9—C8—H8	103.8	N8—C39—C40	110.1 (3)
C1—C8—H8	103.8	N8—C39—H39A	109.6
C12—C9—C10	119.5 (3)	C40—C39—H39A	109.6
C12—C9—C8	123.3 (3)	N8—C39—H39B	109.6
C10—C9—C8	116.7 (3)	C40—C39—H39B	109.6
O2—C10—C9	125.3 (3)	H39A—C39—H39B	108.1
O2—C10—N1	117.7 (3)	C39—C40—H40A	109.5
C9—C10—N1	117.1 (3)	C39—C40—H40B	109.5
N1—C11—N2	116.0 (3)	H40A—C40—H40B	109.5
N1—C11—S1	121.9 (3)	C39—C40—H40C	109.5
N2—C11—S1	122.0 (3)	H40A—C40—H40C	109.5
O1—C12—C9	123.8 (3)	H40B—C40—H40C	109.5
O1—C12—N2	116.1 (3)	C42—C41—C44	119.3 (3)
C9—C12—N2	120.0 (3)	C42—C41—C32	123.0 (3)
N1—C13—C14	110.4 (3)	C44—C41—C32	117.4 (3)
N1—C13—H13A	109.6	O8—C42—C41	125.5 (3)
C14—C13—H13A	109.6	O8—C42—N9	115.2 (3)
N1—C13—H13B	109.6	C41—C42—N9	119.3 (3)
C14—C13—H13B	109.6	N9—C43—N10	116.3 (3)
H13A—C13—H13B	108.1	N9—C43—S4	121.4 (3)
C13—C14—H14A	109.5	N10—C43—S4	122.3 (3)
C13—C14—H14B	109.5	O9—C44—N10	117.0 (3)
H14A—C14—H14B	109.5	O9—C44—C41	126.0 (3)
C13—C14—H14C	109.5	N10—C44—C41	117.0 (3)
H14A—C14—H14C	109.5	N9—C45—C46	113.5 (3)
H14B—C14—H14C	109.5	N9—C45—H45A	108.9
N2—C15—C16	111.4 (3)	C46—C45—H45A	108.9
N2—C15—H15A	109.3	N9—C45—H45B	108.9
C16—C15—H15A	109.3	C46—C45—H45B	108.9
N2—C15—H15B	109.3	H45A—C45—H45B	107.7
C16—C15—H15B	109.3	C45—C46—H46A	109.5
H15A—C15—H15B	108.0	C45—C46—H46B	109.5
C15—C16—H16A	109.5	H46A—C46—H46B	109.5
C15—C16—H16B	109.5	C45—C46—H46C	109.5
H16A—C16—H16B	109.5	H46A—C46—H46C	109.5
C15—C16—H16C	109.5	H46B—C46—H46C	109.5
H16A—C16—H16C	109.5	N10—C47—C48	112.4 (4)
H16B—C16—H16C	109.5	N10—C47—H47A	109.1
C18—C17—C20	119.3 (3)	C48—C47—H47A	109.1
C18—C17—C8	123.7 (3)	N10—C47—H47B	109.1
C20—C17—C8	117.0 (3)	C48—C47—H47B	109.1
O3—C18—C17	125.9 (3)	H47A—C47—H47B	107.9
O3—C18—N3	114.4 (3)	C47—C48—H48A	109.5
C17—C18—N3	119.7 (3)	C47—C48—H48B	109.5
N4—C19—N3	115.9 (3)	H48A—C48—H48B	109.5
N4—C19—S2	121.8 (3)	C47—C48—H48C	109.5
N3—C19—S2	122.3 (3)	H48A—C48—H48C	109.5
O4—C20—C17	124.7 (3)	H48B—C48—H48C	109.5
O4—C20—N4	117.8 (3)	N11—C49—C50	119.2 (4)
C17—C20—N4	117.4 (3)	N11—C49—H49	120.4
C22—C21—C22'	59.7 (13)	C50—C49—H49	120.4
C22—C21—N3	97.3 (6)	C51—C50—C49	119.3 (5)
C22'—C21—N3	156.6 (13)	C51—C50—H50	120.4
C22—C21—H21A	112.3	C49—C50—H50	120.4
C22'—C21—H21A	83.2	C52—C51—C50	119.2 (4)
N3—C21—H21A	112.3	C52—C51—H51	120.4
C22—C21—H21B	112.3	C50—C51—H51	120.4
C22'—C21—H21B	76.0	C51—C52—C53	120.5 (4)
N3—C21—H21B	112.3	C51—C52—H52	119.8
H21A—C21—H21B	109.9	C53—C52—H52	119.8
C22—C21—H21C	115.2	N11—C53—C52	118.9 (4)
C22'—C21—H21C	97.2	N11—C53—H53	120.5
N3—C21—H21C	97.2	C52—C53—H53	120.5
H21B—C21—H21C	119.0	N12—C54—C55	118.7 (7)
C22—C21—H21D	136.2	N12—C54—H54	120.7
H21C—C21—H21D	103.6	C55—C54—H54	120.7
C21—C22—H22A	109.5	C54—C55—C56	120.4 (8)
C21—C22—H22B	109.5	C54—C55—H55	119.8
C21—C22—H22C	109.5	C56—C55—H55	119.8
C21—C22'—H22D	109.5	C55—C56—C57	121.7 (6)
C21—C22'—H22E	109.5	C55—C56—H56	119.1
H22D—C22'—H22E	109.5	C57—C56—H56	119.1
C21—C22'—H22F	109.5	C58—C57—C56	116.8 (6)
H22D—C22'—H22F	109.5	C58—C57—H57	121.6
H22E—C22'—H22F	109.5	C56—C57—H57	121.6
N4—C23—C24	112.3 (4)	C57—C58—N12	119.2 (6)
N4—C23—H23A	109.2	C57—C58—H58	120.4
C24—C23—H23A	109.2	N12—C58—H58	120.4
N4—C23—H23B	109.2		
			
C7—O5—C2—C3	22.1 (7)	C32—C25—C26—C27	178.5 (3)
C7—O5—C2—C1	−159.9 (5)	O10—C26—C27—C28	175.3 (4)
C6—C1—C2—O5	−177.4 (3)	C25—C26—C27—C28	−1.7 (6)
C8—C1—C2—O5	−1.3 (5)	C26—C27—C28—C29	0.2 (7)
C6—C1—C2—C3	0.7 (5)	C27—C28—C29—C30	1.0 (8)
C8—C1—C2—C3	176.7 (3)	C26—C25—C30—C29	−0.8 (6)
O5—C2—C3—C4	177.9 (4)	C32—C25—C30—C29	−177.2 (4)
C1—C2—C3—C4	0.0 (6)	C28—C29—C30—C25	−0.6 (7)
C2—C3—C4—C5	−0.3 (6)	C30—C25—C32—C41	10.4 (5)
C3—C4—C5—C6	−0.1 (6)	C26—C25—C32—C41	−166.0 (3)
C4—C5—C6—C1	0.9 (6)	C30—C25—C32—C33	−124.6 (4)
C2—C1—C6—C5	−1.1 (5)	C26—C25—C32—C33	59.0 (4)
C8—C1—C6—C5	−176.9 (3)	C41—C32—C33—C34	−87.5 (4)
C6—C1—C8—C17	11.1 (4)	C25—C32—C33—C34	46.6 (4)
C2—C1—C8—C17	−164.6 (3)	C41—C32—C33—C36	100.8 (4)
C6—C1—C8—C9	−124.6 (3)	C25—C32—C33—C36	−125.1 (3)
C2—C1—C8—C9	59.7 (4)	C36—C33—C34—O6	−178.0 (3)
C17—C8—C9—C12	−88.5 (4)	C32—C33—C34—O6	10.4 (6)
C1—C8—C9—C12	46.6 (4)	C36—C33—C34—N7	1.9 (5)
C17—C8—C9—C10	99.2 (3)	C32—C33—C34—N7	−169.6 (3)
C1—C8—C9—C10	−125.7 (3)	C35—N7—C34—O6	−179.1 (3)
C12—C9—C10—O2	176.2 (3)	C37—N7—C34—O6	7.9 (5)
C8—C9—C10—O2	−11.1 (5)	C35—N7—C34—C33	0.9 (6)
C12—C9—C10—N1	−4.2 (5)	C37—N7—C34—C33	−172.1 (4)
C8—C9—C10—N1	168.5 (3)	C34—N7—C35—N8	−1.7 (6)
C11—N1—C10—O2	−179.4 (3)	C37—N7—C35—N8	171.1 (4)
C13—N1—C10—O2	6.3 (5)	C34—N7—C35—S3	−179.7 (3)
C11—N1—C10—C9	0.9 (5)	C37—N7—C35—S3	−6.8 (5)
C13—N1—C10—C9	−173.3 (3)	C36—N8—C35—N7	−0.3 (6)
C10—N1—C11—N2	3.3 (5)	C39—N8—C35—N7	−175.9 (4)
C13—N1—C11—N2	177.3 (3)	C36—N8—C35—S3	177.6 (3)
C10—N1—C11—S1	−177.9 (3)	C39—N8—C35—S3	2.1 (5)
C13—N1—C11—S1	−3.9 (5)	C34—C33—C36—O7	177.8 (4)
C12—N2—C11—N1	−4.5 (5)	C32—C33—C36—O7	−10.0 (6)
C15—N2—C11—N1	−178.0 (3)	C34—C33—C36—N8	−3.7 (5)
C12—N2—C11—S1	176.7 (3)	C32—C33—C36—N8	168.4 (3)
C15—N2—C11—S1	3.3 (5)	C35—N8—C36—O7	−178.4 (4)
C10—C9—C12—O1	−175.8 (3)	C39—N8—C36—O7	−2.7 (5)
C8—C9—C12—O1	12.0 (5)	C35—N8—C36—C33	3.0 (6)
C10—C9—C12—N2	3.1 (5)	C39—N8—C36—C33	178.7 (3)
C8—C9—C12—N2	−169.1 (3)	C35—N7—C37—C38	−87.8 (5)
C11—N2—C12—O1	−179.5 (3)	C34—N7—C37—C38	85.4 (5)
C15—N2—C12—O1	−5.9 (5)	C35—N8—C39—C40	85.1 (5)
C11—N2—C12—C9	1.5 (5)	C36—N8—C39—C40	−90.8 (4)
C15—N2—C12—C9	175.1 (3)	C25—C32—C41—C42	−70.0 (4)
C11—N1—C13—C14	−93.3 (4)	C33—C32—C41—C42	64.1 (4)
C10—N1—C13—C14	81.2 (4)	C25—C32—C41—C44	104.6 (3)
C11—N2—C15—C16	86.2 (4)	C33—C32—C41—C44	−121.4 (3)
C12—N2—C15—C16	−87.6 (4)	C44—C41—C42—O8	−178.6 (4)
C9—C8—C17—C18	59.2 (5)	C32—C41—C42—O8	−4.1 (6)
C1—C8—C17—C18	−74.9 (5)	C44—C41—C42—N9	−0.6 (5)
C9—C8—C17—C20	−119.8 (3)	C32—C41—C42—N9	173.9 (3)
C1—C8—C17—C20	106.1 (4)	C43—N9—C42—O8	−175.8 (3)
C20—C17—C18—O3	−177.9 (5)	C45—N9—C42—O8	5.7 (5)
C8—C17—C18—O3	3.1 (7)	C43—N9—C42—C41	6.0 (5)
C20—C17—C18—N3	1.1 (7)	C45—N9—C42—C41	−172.4 (3)
C8—C17—C18—N3	−177.9 (4)	C42—N9—C43—N10	−2.7 (5)
C19—N3—C18—O3	−179.2 (6)	C45—N9—C43—N10	175.7 (3)
C21—N3—C18—O3	10.1 (8)	C42—N9—C43—S4	176.9 (3)
C19—N3—C18—C17	1.7 (9)	C45—N9—C43—S4	−4.7 (5)
C21—N3—C18—C17	−169.0 (5)	C44—N10—C43—N9	−6.1 (5)
C20—N4—C19—N3	2.3 (7)	C47—N10—C43—N9	178.4 (3)
C23—N4—C19—N3	−176.8 (5)	C44—N10—C43—S4	174.3 (3)
C20—N4—C19—S2	−178.1 (3)	C47—N10—C43—S4	−1.2 (5)
C23—N4—C19—S2	2.8 (7)	C43—N10—C44—O9	−171.1 (3)
C18—N3—C19—N4	−3.3 (9)	C47—N10—C44—O9	4.5 (5)
C21—N3—C19—N4	167.1 (5)	C43—N10—C44—C41	11.1 (5)
C18—N3—C19—S2	177.1 (5)	C47—N10—C44—C41	−173.3 (3)
C21—N3—C19—S2	−12.4 (9)	C42—C41—C44—O9	175.1 (4)
C18—C17—C20—O4	−179.8 (4)	C32—C41—C44—O9	0.3 (5)
C8—C17—C20—O4	−0.7 (6)	C42—C41—C44—N10	−7.3 (5)
C18—C17—C20—N4	−2.0 (6)	C32—C41—C44—N10	177.9 (3)
C8—C17—C20—N4	177.1 (3)	C43—N9—C45—C46	89.2 (4)
C19—N4—C20—O4	178.2 (4)	C42—N9—C45—C46	−92.3 (4)
C23—N4—C20—O4	−2.6 (6)	C43—N10—C47—C48	87.6 (5)
C19—N4—C20—C17	0.3 (6)	C44—N10—C47—C48	−88.2 (4)
C23—N4—C20—C17	179.4 (4)	C53—N11—C49—C50	0.8 (2)
C19—N3—C21—C22	96.0 (7)	N11—C49—C50—C51	0.4 (2)
C18—N3—C21—C22	−92.9 (6)	C49—C50—C51—C52	−0.7 (4)
C19—N3—C21—C22'	85 (3)	C50—C51—C52—C53	−0.2 (5)
C18—N3—C21—C22'	−104 (3)	C49—N11—C53—C52	−1.6 (4)
C19—N4—C23—C24	−85.3 (5)	C51—C52—C53—N11	1.3 (5)
C20—N4—C23—C24	95.5 (5)	C58—N12—C54—C55	−0.5 (3)
C31—O10—C26—C25	−169.0 (3)	N12—C54—C55—C56	0.3 (3)
C31—O10—C26—C27	13.9 (5)	C54—C55—C56—C57	−0.8 (6)
C30—C25—C26—O10	−175.2 (3)	C55—C56—C57—C58	1.5 (7)
C32—C25—C26—O10	1.4 (5)	C56—C57—C58—N12	−1.6 (6)
C30—C25—C26—C27	2.0 (5)	C54—N12—C58—C57	1.2 (5)

### Hydrogen-bond geometry (Å, °)

**Table:**

*D*—H···*A*	*D*—H	H···*A*	*D*···*A*	*D*—H···*A*
O3—H3···O1	0.84	1.63	2.435 (4)	159
O6—H6···O8	0.84	1.67	2.444 (4)	152
N11—H11···O2	0.88	1.93	2.721 (4)	148
N12—H12···O9	0.88	1.89	2.745 (6)	162
